# Endocrine Disorders in Critically Ill Patients – The Smooth Criminal?

**DOI:** 10.2478/jccm-2024-0034

**Published:** 2024-07-31

**Authors:** Bianca-Liana Grigorescu, Raluca Ștefania Fodor

**Affiliations:** George Emil Palade University of Medicine, Pharmacy, Science, and Technology of Targu-Mures, Romania

The hallmark of critical illness is the rapid initiation of numerous physiologic processes in an effort to reestablish homeostasis. Thus, critical illness can be considered the result of an acute physical stress that acts as a trigger for an acute aggressive inflammatory response [1,2].

The inflammatory response occurs early after primary injury, almost concomitant with coagulation cascade activation, micro- and macrovascular alterations, and hypothalamic-pituitary-peripheral axis disorders, proportionate to the severity of illness. In this context, “The Triad of Critically Illness” is a lethal interplay involving three key factors: inflammation, coagulopathy and hormonal imbalance.

Clinical practice in ICU is directed mainly towards advanced respiratory and hemodynamic support, renal impairment or neurological alterations, than to endocrine homeostasis. Hence, the intensivist is faced to clinical “unexplainable” situations (resistant hyperglycemia, prolonged hypercatabolic status, refractory hypotension despite adequate volume resuscitation or vasopressors administration), since the neuroendocrine disorders are nonspecific and “silent” [3].

The stress response was the outcome of a multitude of intricately linked neural and endocrine responses that were designed to ensure cellular energy and hemodynamic stability during the acute phase of critical illness [2].

Similar to the inflammatory response, the hormonal stress response follows a biphasic pattern. The first phase, or “acute phase” starts minutes or hours after the occurrence of the event. The release of large amounts of proinflammatory cytokines, excessive activation of sensory neurons and catecholamine release trigger the “fight or flight” state. Cellular hypoxia or reduced oxygen supply and impaired activation of defensive mechanisms are the primary factors contributing to mitochondrial dysfunction, which is the leading cause of multiple organ failure. The acute phase is characterized by the attenuation of neuroendocrine axis, leading to a complex alteration in peripheral hormone levels, secondary to a decrease in nutritional intake and a shift towards a catabolic state [2].

The second phase or “chronic phase” of the stress response is marked by uniform suppression of all neuroendocrine axes, secondary to the high serum levels of cytokines, improved nutritional signals, and a decrease in hypothalamic hypophysiotropic hormones [2].

The five main neuroendocrine axes involved in the neural and endocrine response in critical ill patients are the somatotropic axis, the thyroid axis, the adrenal axis, the lactotropic axis, and the gonadal axis.

The somatotropic axis: Growth hormone (GH) serum concentrations start to rise hours after the onset of critical illness. The pulsatile pattern of GH secretion diminishes and the peak of GH becomes less pronounced, while levels of insulin-like growth factor-1 (IGF-1), IGF-binding protein 3 (IGFBP-3), and acid label subunit (ALS) remain low. During the acute phase, there is a development of resistance to GH in the peripheral tissues. This resistance is caused by the suppression of GH receptors in the liver, leading to a change from the anabolic effects of IGF-1 to the more catabolic actions of GH. Hepatic GH resistance is reduced throughout the chronic stage of severe disease. The persistence of high serum levels of GH accounts for toxic side effects such as excessive fluid retention, hypercalcemia and pronounced insulin resistance with hyperglycemia [2].

The thyroid axis: The inflammatory response of critical illness also interferes with the homeostasis of thyroid hormones. At least two-thirds of critically ill patients without preexisting thyroid disease and without hypothalamic-pituitary axis dysfunction exhibit typical changes in thyroid economy known by various names, including Nonthyroidal Illness Syndrome (NTIS), Low T3 Syndrome, or Sick Euthyroid Syndrome [4]. This results in a reduction of circulating thyroid hormone levels, but without obvious signs or symptoms of hypothyroidism. The pathophysiological mechanism underlying the variation of thyroid hormones in critical illness has not yet been fully elucidated, but it is attributed to a combination of cytokine inflammatory activity, which disrupts the central and peripheral regulation of thyroid hormones, and caloric restriction, which decreases the level of leptin, the hunger hormone [5].

Although, the degree of thyroid dysfunction seems to correlate with disease severity and prognosis in critically ill patients [6, 7], the administration of thyroxine to these patients has not provided additional benefits. Therefore, the Nonthyroidal Illness Syndrome might be a self-protective mechanism of the body by lowering the basal metabolic rate and limiting excessive catabolism. Thyroid function can be fully restored after recovering from the non-thyroidal illness that caused the syndrome [8]. Prolonged critical illness can lead to the development of transitory central hypothyroidism, which is characterized by a notable reduction in T3, thyroxine (T4), and TSH, as well as elevated levels of rT3. In the absence of thyroid hormone replacement therapy, this condition could impede recovery [9]. Patients with serum TSH concentrations above 20 mU/L usually have permanent hypothyroidism [10]. Central hypothyroidism may exist in critically ill patients and should be taken seriously.

The adrenal axis is characterized by elevated levels of the stress hormone cortisol in the bloodstream, accompanied by the disruption of the normal daily patterns of ACTH and cortisol. Albumin and cortisol-binding proteins (CBG) are suppressed, leading to an additional increase in free cortisol serum levels [2]. During the “fight or flight” phase, the significantly increased levels of cortisol play a crucial role in regulating the immunological response, improving blood flow, and supplying energy. Critical illness-related corticosteroid insufficiency (CIRCI) was described as the impairment of the hypothalamic-pituitary axis during critical illness [11]. The signs and symptoms of CIRCI are non-specific and comprise adverse neurological recovery after the cessation of sedation, encephalopathy, electrolyte disturbances (hypercalcemia and hyponatremia), eosinophilia and unexplained hemodynamic instability, and increased bile acids and bilirubin serum levels [12].

The lactotropic axis: Patients with sepsis or septic shock presented high serum levels of prolactin (PRL) in the first days after the acute injury onset. In the prolonged chronic phase of critical illness PRL serum levels decrease to suppression.

The gonadal hormones: are severely decrease in critical ill patients. Research indicates that administering oxandrolone, a synthetic androgenic steroid with anabolic properties, to patients with severe burns can decrease the duration of their hospital length of stay due to its ability to stimulate muscle growth-the anabolic response [1,2].

Neuroendocrine alterations in in the general ICU population: acute respiratory distress syndrome (ARDS) patients exhibit a higher hormonal response, different than acute neurocritical patients. The magnitude of hormonal response is higher, secondary to thyroid axis suppression and alterations in prolactin and copeptin serum levels. Activation of the hypothalamic-pituitary-adrenal axis (HPA) is responsible to a higher average in cortisol levels. Differences in central and peripheral thyroid axis function between acute and prolonged critical illness are connected to the extent of the FT4 decline, which correlates with illness severity and outcomes. An increase in copeptin levels indicates the stress response and serves as a prognostic tool in sepsis, traumatic brain injury (TBI), and subarachnoid hemorrhage (SAH). Patients with ARDS demonstrate the most pronounced activation of copeptin, as well as reduced levels of IGF1, activation of prolactin, and suppression of the thyroid axis. This combination results in a more vigorous stress response compared to patients with neurological conditions. Nutritional and hormonal state, as well as body weight, can affect GH/IGF1 levels, which in turn can have an impact on the release of GH in the pituitary gland [13].

Neuroendocrine alterations in neurocritical ill patients: acute neurological patients are more likely to develop pituitary deficit as a result of initial damage or secondary insults such low blood pressure, low oxygen levels, or intracranial hypertension. Brain death (BD) is characterized by widespread and severe lack of blood flow, resulting in pituitary dysfunction, unstable blood pressure, and degeneration of organs [14]. Functional alterations of the posterior pituitary gland can be evaluated by assessing the copeptin levels. Copeptin, a component of the Arginine Vasopressin (AVP) precursor, serves two primary functions: as a diagnostic indicator for diabetes insipidus (when its concentrations are reduced) and as a biomarker for stress response (when its concentrations are elevated). Copeptin activation is observed in 60–80% of SAH and TBI cases. The activation of all stress-related hormone axes occurs during the early phase after TBI, while anabolic hormones like thyroid and IGF1 are inhibited. When comparing TBI to SAH, SAH had a lesser occurrence of thyroid axis suppression but a larger presence of copeptin and activation of the HPA axis. Suppression of the thyroid axis reduce the vascular response to vasopressin, leading to refractory hypotension. Activation of the HPA axis may mitigate the effects of proinflammatory cytokines following brain death [14, 15].

Although endocrine dysfunction in critically ill patients is a topic we likely encounter daily at the patient’s bedside, many intensivists accustomed to invasive maneuvers and procedures, may forget to consider it. Intensivist should be aware that critical illness is a deadly game of three players: inflammation, coagulopathy and hormonal imbalance.

## References

[1] Langouche L, Teblick A, Gunst J, Van den Berghe G (2023). The hypothalamus-pituitary-adrenocortical response to critical illness: a concept in need of revision. Endocrine Reviews.

[2] Teblick A, Langouche L, Van den Berghe G (2019). Anterior pituitary function in critical illness. Endocrine Connections.

[3] Siddaqui SS, Dominic N, Kumar S (2022). Diagnosis of Sheehan’s syndrome in non-obstetric critical care and emerging settings: a case series of five patients with varied presentations. J Crit Care Med (Targu-Mures).

[4] Van den Berghe G (2014). Non-thyroidal illness in the ICU: a syndrome with different faces. Thyroid.

[5] Fliers E, Bianco AC, Langouche L, Boelen A (2015). Thyroid function in critically ill patients. Lancet Diabetes Endocrinol.

[6] Rothberger GD, Gadhvi S, Michelakis N (2017). Usefulness of Serum Triiodothyronine (T3) to Predict Outcomes in Patients Hospitalized With Acute Heart Failure. Am J Cardiol.

[7] Liu J, Wu X, Lu F (2016). Low T3 syndrome is a strong predictor of poor outcomes in patients with community-acquired pneumonia. Sci Rep.

[8] Silveira CDG, de Vasconcelos FPJ, Moura EB, da Silveira BTG, Amorim FFP, Shintaku LS (2021). Thyroid Function, Reverse Triiodothyronine, and Mortality in Critically Ill Clinical Patients. Indian J Crit Care Med.

[9] Bello G., Pennisi M.A., Montini L. (2009). Nonthyroidal illness syndrome and prolonged mechanical ventilation in patients admitted to the ICU. Chest.

[10] Douglass Ross Thyroid function in nonthyroidal illness.

[11] Annane D, Pastores SM, Rochwerg B (2017). Guidelines for the diagnostic and management of critical illness-related corticosteroid insufficiency (CIRCI) in critically ill patients (Part I):Society of Critical Care Medicine (SCCM)and European Society of Intensive Care Medicine (ESCIM)2017. Critical Care Medicine.

[12] Teblick A, Gunst J, Van den Berghe G (2022). Critical illness-induced corticosteroid insufficiency:what it is not and what it could be. J Clin Endocrinol Metab.

[13] Mazzeo AT, Guaraldi F, Fillipini C (2019). Activation of pituitary axis according to underlying critical illness and its effect on outcome. J Crit Care.

[14] Grigorescu BL (2020). Hypothalamic-pituitary axis disorder- “the puppet master” of multiple organ dysfunction in brain-death patients. J Crit Care Med (Targu-Mures).

[15] Mahajan C, Prabhakar H, Bilotta F (2023). Endocrine dysfunction after traumatic brain injury: an ignored clinical syndrome?. Neurocrit Care.

